# Organochlorine pesticide residues in plants and their possible ecotoxicological and agri food impacts

**DOI:** 10.1038/s41598-021-97286-4

**Published:** 2021-09-08

**Authors:** Rachna Chandra, N. Sharpanabharathi, B. Anjan Kumar Prusty, P. A. Azeez, Rama Mohan Kurakalva

**Affiliations:** 1 Gujarat Institute of Desert Ecology (GUIDE), P.B. # 83, Mundra Road, Bhuj, 370001 Gujarat India; 2 Present Address: Department of Environmental Sciences - Forestry, Faculty of Agriculture, Sri Sri University, Sri Sri Vihar, Godi Sahi, Cuttack, 754006 Odisha India; 3 Sálim Ali Centre for Ornithology and Natural History (SACON), Anaikatti (PO), Coimbatore, 641108 Tamil Nadu India; 4 Department of Natural Resources Management and Geoinformatics, Khallikote University, Berhampur, 761008 Odisha India; 5 Society for Sustainable Systems, Kovaipudur (PO), Coimbatore, 641042 Tamil Nadu India; 6 Hydrogeochemistry Group, CSIR – National Geophysical Research Institute (NGRI), Uppal Road, Hyderabad, 500007 Telangana India; 7 Academy of Scientific & Innovative Research (AcSIR), Ghaziabhad, 201002 India

**Keywords:** Environmental sciences, Health care

## Abstract

Scientific investigations on levels of Organochlorine Pesticide (OCP) residues in plants largely consider the edible parts (crops, vegetables, and fruit plants). Though the non-edible parts of plants are not eaten by human beings directly, these parts are consumed by livestock and other animals, thereby facilitating the flow of chemical residues through the food chain. The objective of the present investigation was to evaluate the concentration of OCP residues in non-edible plant parts to provide insights on their potential ecotoxicological impacts. Eighteen OCP residues were extracted in nine different plant species (banana *Musa acuminate,* brinjal *Solanum melongena, Casuarina equisetifolia*, *Eucalyptus globulus*, lotus *Nelumbo nucifera*, paddy *Oryza sativa*, sugarcane *Saccharum officinarum*, tapioca *Manihot esculenta*, tomato *Lycopersicon esculentum*) following QuEChERS method. The concentrations of OCP residues in plant extracts were determined using Gas Chromatography coupled with Mass Spectrometry (GC–MS). The OCP residues, namely: *γ*-HCH (lindane), heptachlor epoxide isomer, dieldrin, endrin, endrin aldehyde and endrin ketone were found predominantly in seven plant species. Residues of γ-HCH (lindane) were reported in different parts of plant species such as stem (581.14 ng/g in paddy and 585.82 ng/g in tapioca) and leaf (583.3 ng/g in tomato). Seven samples contained residues of heptachlor epoxide isomer (512.53 to 1173.8 ng/g). Dieldrin was found in paddy stem (489.97 ng/g), tapioca stem (490.21 ng/g) and tapioca leaf (490.32 ng/g). The detected OCPs in the present study were 10–50 times higher than the Maximum Residue Limits (MRL, 0.01–0.1 mg/Kg) as prescribed in the Codex Alimentarius of the FAO/WHO. Their elevated concentrations in the plant parts therefore pose risk of contamination to the consumers in the food chain, including human beings those are dependent on the animals as source of protein. The findings of this study are the first report on residue levels of OCPs in non-edible plant parts in the agricultural landscape of Puducherry region, India. Since, this study assumes significance for the strategic location of Oussudu Lake, an interstate lake spread over Puducherry and Tamil Nadu states, regular monitoring of OCP residues in different environmental segments in strategic locations in both the states is suggested, which will help the authorities in devising a comprehensive environmental management plan aiming at the ecosystem at large.

## Introduction

Intensification of agriculture has been a common scenario worldwide, which invariably aims at producing better quality crops that can be both cost effective and sustainable. Such intensive agricultural activities regularly make use of various agrochemicals, i.e., chemical fertilizers and pesticides^1–3^, and in India, the application of such agrochemicals has been beyond the dosage^4^ recommended by the agencies such as Indian Council for Agricultural Research (ICAR). Reports of inappropriate dosage of chemical pesticides in farming systems are also found in different parts of the globe^5^. Since the 1970s, rapid growth in the manufacture and use of chemical pesticides and their toxic effects have raised concerns about ecological and human health impacts. About 64% of global agricultural land (approximately 24.5 million km^2^) is at the risk of pesticide contamination^6^ due to more than one active ingredient, and 31% of agricultural land is at high risk. Furthermore, the watersheds in countries such as South Africa, China, India, Australia and Argentina are classified as high-concern regions as they have high pesticide contamination risk, bear high biodiversity and suffer from water scarcity. The pathways for such agrochemicals in the ecosystem and their potential impact on ecosystem have been extensively studied^2^. Upon application of agrochemicals onto the crops, pesticides may interact with the plant surfaces, or be exposed to the environmental factors such as wind and sun, and may be washed off during rainfall. Pesticides may be absorbed by the plant surface (waxy cuticle and root surfaces) and enter the plant transport system (systemic) or stay on the surface of the plant (contact)^7^. When the pesticides are on the crop surface, they can undergo various processes such as volatilization^8^, photolysis, chemical and microbial degradation^9^. All these processes eventually reduce the original pesticide concentration but can also introduce some metabolites in the crops. Rain wash-off can be important when it occurs shortly after application, and such processes have toxicological implications for receiving water bodies.

In the past half century, yields have either ceased to increase or even declined by 38.8% in several major cereal-growing areas across the globe, and India is no exception to this^4^. Chemical pesticides are often target chemicals used to control specific types of pests^10^ in various crops^11,12^. The use of organochlorine pesticides (OCPs) has significantly increased crop yields, and its deleterious effects on the environment are well reported^13,14^. Organochlorine pesticides have the potential to bioaccumulate and bio-magnify via their movement/flow along the food chain^15^. Accumulation of OCPs in root vegetables has been reportedly greater than other type of vegetables^16,17^. High persistence, low polarity, low aqueous solubility and high lipophilicity are some of the significant features of OCPs which make them a preferred chemical group for extensive use in pest and vector control in India. Moreover, the physico-chemical properties and persistent nature of OCPs in the environment make them ideal for long-range transport^18^. A range of variables such as low decomposition rate, longer half-life, and persistent toxicity allow pesticides to remain in the environment for longer periods resulting in adverse effects on non-target species. Despite the ban on and restriction in the use of OCPs, several developing countries are still using them to mitigate pests in agricultural sector and for public health purposes, because of their lower cost and flexibility in pest control^19^. Developing countries experience an annual death of around 20,000 people due to pesticide poisoning through food^20^. Hence, there has been widespread concern about the long-term effects of chemicals used in India for agricultural purposes.

During the last few decades, the transition from conventional farming to chemical based farming system in India has been quite apparent with almost all the farming practices making intensive use of agrochemicals. Thus, over the years use of a variety of chemical pesticides in agricultural practices has led to its flow through the ecosystem, and bioaccumulation in different food products such as fruits, vegetables and grains. Basically, many of the scientific investigations focused on determination of pesticide residue levels in fruits, vegetables^21–29^ and grains^30–32^ and many of the earlier studies have examined the level of OCP residues in the edible plant parts^14,24,30^ that are directly consumed by human, whereas the crop parts that remain unconsumed by human beings (such as roots, leaves, stems) are scarcely studied^14^. Such non-edible parts of plants are either fed to the livestock as a routine or left as such in farmlands as a nutrient source for next crop cycle. The ingestion of crops/plants by livestock carries a risk of biomagnification and its eventual transfer to human and other organisms occupying next order trophic stages in the ecosystem. In this context, the present work focuses on the evaluation of OCP residues in non-edible parts of plants and its possible implications on ecosystem from different villages in and around Oussudu Lake, Puducherry region, India.

The present study, in and around Oussudu Lake, Puducherry region, assumes significance for the ecological importance of the lake, and dependence of surrounding villagers on the lake for irrigation, drinking, bathing and washing of clothes. Oussudu Lake, the largest wetland in Puducherry region, is a natural habitat for several migratory bird species^33^. This Lake is ranked as one of the important wetlands in Asia, and identified as a world heritage site by International Union of Conservation of Nature (IUCN). Topographically, the lake is in a state of ecological equilibrium with the surrounding villages for its strategic location and thus affected by the human activities. About 50% of the villages in the area depend on the lake water for irrigation purpose^34^. Over the past decades all the villages have shifted to chemical based farming practices involving use of various chemical fertilizers and pesticides, presumably facilitating an enhancement in the chemical residue levels in the lake. In view of these specifics, the present investigation was aimed at evaluating the concentration of OCP residues in nonedible parts of plants and establishing their ecotoxicological effects.

## Materials and methods

### Study area

The area chosen for the proposed study covered 22 villages around Oussudu lake, Puducherry region, India. The lake is located within the latitudes 11° 56ʹ–11° 58ʹ N and longitudes 79° 44ʹ–79° 45ʹ E at a distance of 10 km from Puducherry town in the Western side on Puducherry-Villupuram-Valuthavur main road. The Oussudu Lake, a wetland of national importance^35^, is spread across Puducherry (390 ha) and Tamil Nadu (around 510 ha) states^36^. Rural settlements predominate along the state of Tamil Nadu side of the lake shore and industrial, urban and suburban areas are mostly seen on Puducherry side^34^, which are likely to impact the lake and its surrounding settlements. The marine, fluvial and fluvio-marine regimes shape the landforms in the region that support individual soil assemblies. Oussudu lake watershed comprises mostly of alluvium, Manaveli clay stone, and Vanur sandstone. The climate of the area is humid and tropical with an annual rainfall of around 1200 mm, of which 63% falls during monsoon. The mean temperature ranges from 28 °C in winter to 39 °C in summer^36^. Agriculture is the major land use followed by settlements and plantations. Plantations in the catchment area of the Oussudu Lake are primarily of *Casuarina sp*. and coconut, occupying 1192 ha. The vegetation of the area consists of herbs, shrubs, very large trees and aquatic plants. Around 480 plant species, belonging to 317 genera and 92 families, have been recorded from the area^34,36^. During the survey, a large-scale cultivation of paddy was perceived in the region. Coconut farms were also very common around the lake. In addition to paddy, sugarcane, *Casuarina*, cashew nut and vegetables are also grown in the area.

### Preliminary survey

A preliminary survey was conducted to understand the farming practices and trends in the region. The survey was carried out in 22 villages (upstream and downstream) using a tailor-made questionnaire with both close and open-ended questions. The questionnaire included several parameters such as farming practices and types of agro-chemicals used nearby water bodies, dependence on nearby water bodies for agriculture, source of irrigation, method of irrigation, cropping pattern, knowledge on agro-chemicals (type, quantity and mode of application, crop-wise application rate, frequency of application), use of bio-fertilizers, infestation of pests/weeds, cropping pattern and common crop pests, and so on. Attempts were also made to obtain information on the shift in cropping practice, if any, over the last 30 years. Based on the information collected, the villages both on upstream and downstream of the lake were screened, and subsequently the sampling stations were fixed. No human being was harmed and surveys were carried out in accordance with relevant guidelines and regulations. Further, experimental protocols were approved by the Internal Research Committee of Sálim Ali Center for Ornithology and Natural History (SACON), Coimbatore, India. The informed consent for the participation in the study was taken from the villagers prior to the questionnaire survey. No person under 18 years formed part of the survey.

### Sampling and sample preparation

**Figure 1:**
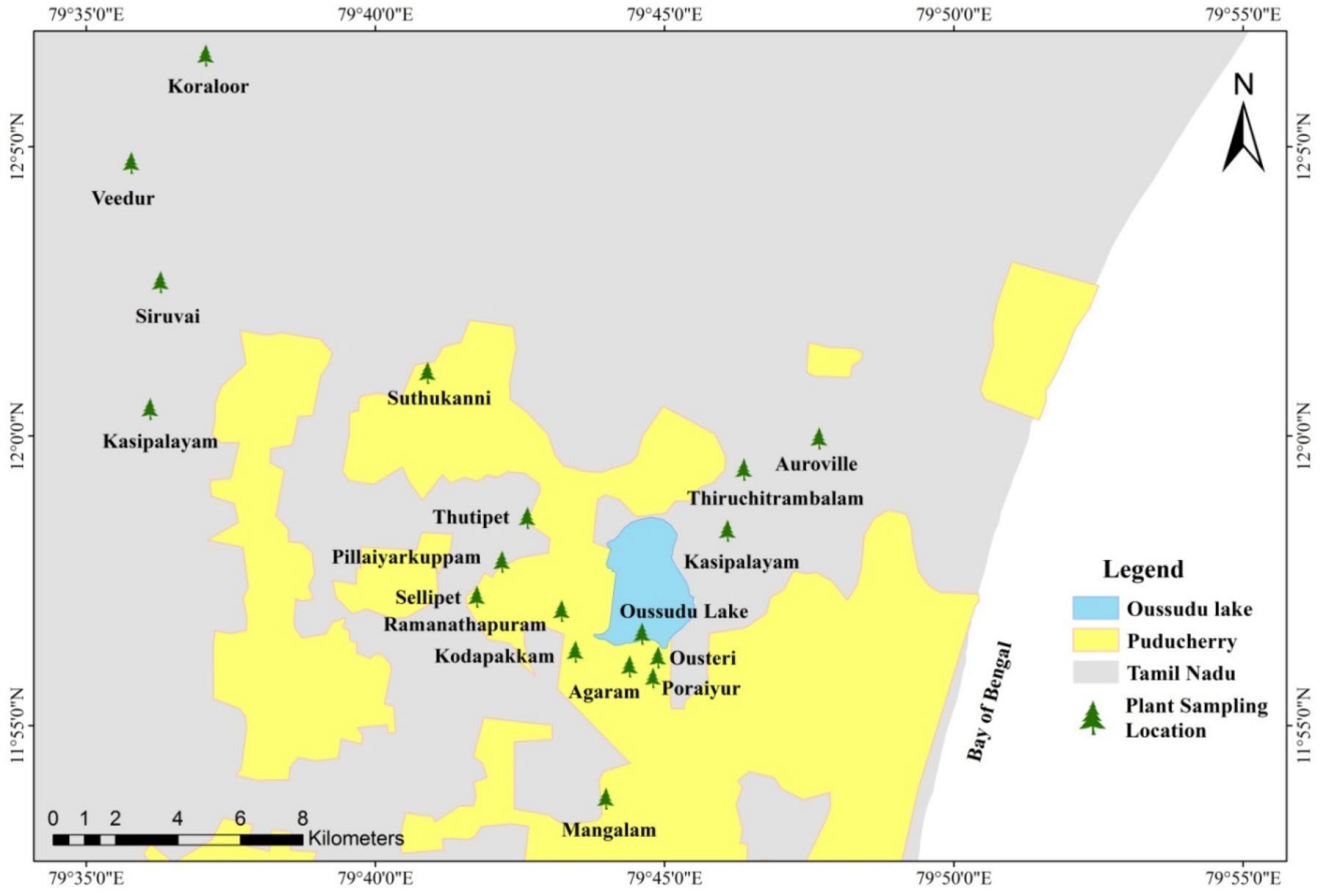
Geographical location map and plant sampling stations in and around Oussudu Lake, Puducherry region (prepared using QGIS version 3.16).

Plant samples were collected and packed in pre-cleaned air-tight polyethylene bags from the established sampling stations in and around Oussudu lake and transported to the laboratory. The geographical location of the study area and sampling locations are marked in Fig. 1. The study area map was prepared using the software QGIS (version 3.16). The boundary of the Oussudu lake was extracted from the dataset of IBA of India provided by Birdlife international^37^. The formal identification of the collected plant samples, namely: paddy (*Oryza sativa:* stem, leaves, grain), tapioca (*Manihot esculenta*: stem, leaves, fruit), brinjal (*Solanum melongena*: stem, leaves, fruit), sugarcane (*Saccharum officinarum*: stem, leaves, fruit), banana (*Musa* sp.: stem, leaves, fruit), tomato (*Solanum lycopersicum*: stem, leaves), blue gum (*Eucalyptus* sp, stem), and iron wood (*Casuarina* sp. stem and leaves) was undertaken by the corresponding author. Since, the collected plant species are common, no voucher specimen was submitted in a publicly available herbarium. For sample collection, verbal consent and permission was obtained from land owners and farmers within the vicinity of the sampling area. In the laboratory, the plant samples were washed thoroughly under running tap water followed by washing with deionized water. Further, the vegetable samples were cut into root, stem, leaf and fruit/grain following standard procedures^22^, and subjected to the pre-treatment methods and protocols for extracting pesticide residues. In total, 45 samples were analyzed in the present study. The analytical experiments were conducted following standard scientific methods^38^.

### Reagents and chemicals

Certified Reference Materials (CRMs) of OCPs containing 18 OCP residues, namely: *α*-HCH, *β*-HCH, *γ*-HCH (lindane), *δ*-HCH, heptachlor (purified), aldrin, heptachlor epoxide isomer, *α*-chlordane, *γ*-chlordane, 4,4'-DDE, dieldrin, endrin, endosulfan-I (i.e. *α*-endosulfan), 4,4'-DDD, endrin aldehyde, 4,4'-DDT, endrin ketone, and methoxychlor, each at a concentration of 2000 mg/L were purchased from SUPELCO, Bellefonte, PA, USA. Stock CRMs were diluted to prepare calibration standards for use in GC–MS analyses. Solid-phase extraction (SPE) sorbents, primary secondary amine (﻿PSA) were obtained from AGILENT TECHNOLOGIES, USA. HPLC grade solvents (acetonitrile, toluene) were used for sample preparation and analysis. Anhydrous magnesium sulfate, sodium citrate and sodium chloride (analytical reagent grade) were purchased from MERCK LIFE SCIENCE, India. Milli-Q water purification system (MILLIPORE, India) was used to obtain ultrapure deionized water.

### Extraction of OCP residues

Most traditional analytical methods for extracting pesticide residues are relatively expensive, lengthy, and consume more samples as well as solvents and chemicals. Thus, the OCP residues from plant samples were extracted following a modified version of QuEChERS method^22^, an anagram for Quick, Easy, Cheap, Effective, Rugged and Safe. This method involves a single extraction in acetonitrile and requires only a limited quantity (10 g) of sample. This initial step of QuEChERS simultaneously extracts the pesticides from the sample and prepares them for the next stage of dispersive solid-phase extraction (dispersive-SPE) process. Samples for pesticide residues were homogenized and extracted with correct suite of organic solvents according to the European Committee for Standardization (EU CEN) 15,662 modified QuEChERS procedure. This technique has an additional step than the initial QuEChERS method, in which citrate salts are applied to the tube during liquid–liquid partitioning. The salts and SPE sorbents involved in the dispersive-SPE process eliminate the residual water and matrix interferences. The resulting acetonitrile extract was analyzed directly by GC–MS with appropriate dilution. To do this, 10 g of the homogenized sample of the plant parts was placed in a Teflon centrifuge tube (40 mL). Subsequently, 10 mL each of distilled water and acetonitrile (MeCN) were added with the cap firmly screwed and the content shaken vigorously in a Vortex mixer at 3000 rpm for 1 min. Then, 4 g of magnesium sulphate (MgSO_4_), 1 g of sodium chloride (NaCl) and 1.5 g of sodium citrate were added, immediately vortexed for 1 min and centrifuged for 5 min at 5000 rpm. Subsequently, 4 mL of the supernatant was transferred to another set of clean-up (centrifuge) tubes containing 0.1 g of PSA sorbent material and 0.6 g of anhydrous MgSO_4_, capped tightly and shaken for 30 s. The centrifuge tubes containing plant extracts were then centrifuged for 5 min at 5000 rpm to separate the solids from solution. Further, as part of dispersive-SPE cleanup, 0.1 g of graphitized carbon black (GCB) was added to the samples having large amounts of pigments. The resulting extract was concentrated using a rotary evaporator (CYBERLAB-RE 10), reconstituted with toluene and stored in amber vials at 4 °C for further analysis using GC–MS.

### Analytical method

The target compounds in plant extracts, after dispersive-SPE procedure, were identified and quantified using a Gas Chromatograph (Perkin Elmer Clarus500) interfaced with a quadrupole Mass Spectrometer (Clarus500). The GC-qMS facility at the Environmental Geochemistry Lab (EGL), CSIR-National Geophysical Research Laboratory (NGRI), Hyderabad, India was utilized in the present study. The GC-qMS system having a capillary column DB-5MS fused silica column (30.0 m × 0.25 mm, i.d. 0.25 μm film thicknesses) was used for separation of target OCPs. The oven temperature was programmed from 80˚C (initial time, 2 min) to 205˚C at a rate of 30˚C/min (hold time 5 min) and then heated to 290˚C at a rate of 10˚C/min with a final holding time of 3 min. The GC system was run in splitless mode, and an auto sampler was used to inject 1 *μ*L aliquots of extracts. The carrier gas was helium with a steady flow rate of 1.0 mL / min. The energy used to ionize electrons was 70 eV. Chromatographic data were obtained in full scan mode and analyzed using the program TurboMass software (Version 6.1.0, https://www.perkinelmer.com/Content/relatedmaterials/productnotes/prd_clarus500gcmssoftware.pdf). Quantification for individual OCPs was performed using the external calibration and a five-point calibration curve constructed at a range of 5-100 ng/L^38^.

### Quality control and quality assurance

For quality control of the data, duplicate samples, and a spiked sample with standards were analyzed to avoid interference and contamination. The spiked samples were prepared with a concentration of 5 ng/L of OCPs studied. Surrogate standard (4,4'-dichlorobiphenyl, 100 *µ*L, 1* µg* ml^-1^) was used to compensate for losses during sample extraction, and the average recoveries were between 81% and 96% for target pesticides (Table 1). The limit of detection (LOD) of OCPs was determined as the concentration of analyte in a sample that gives rise to a peak with a signal-to-noise ratio (S/N) of 3. The limit of quantification (LOQ) is the concentration at ten times to the signal-to-noise ratio of the baseline (Table 1).

**Table 1:** Mean recovery, relative standard deviation (RSD) and limit of detection (LOD) of OCPs.

Compound	Mean recovery (%)	Precision RSD (%)	LOD (ng/g)	LOQ (ng/g)	Calibration curve, r^2^
*α-HCH*	95	0.5	0.002	0.005	0.9984
*β-HCH*	94	1.5	0.002	0.006	0.9997
*γ-HCH*	88	1.1	0.001	0.005	0.9982
*δ-HCH*	81	1.3	0.001	0.008	0.9995
*Heptachlor*	94	0.3	0.004	0.009	0.9996
*Heptachlor epoxide Isomer*	92	0.1	0.004	0.011	0.9978
α-chlordane	84	1.6	0.002	0.009	0.9968
γ-chlordane	82	1.5	0.001	0.008	0.9975
4,4'-DDT	93	0.9	0.001	0.006	0.9993
4,4'-DDE	89	0.3	0.002	0.006	0.9981
4,4'-DDD	87	0.8	0.002	0.006	0.9979
Aldrin	89	0.3	0.001	0.004	0.9988
Dieldrin	96	0.9	0.001	0.006	0.9991
Endrin	90	0.8	0.003	0.008	0.9985
Endrin Aldehyde	93	0.5	0.002	0.005	0.9973
Endrin Ketone	91	0.4	0.002	0.005	0.9993
Endosulfan-I (α)	85	1.2	0.001	0.007	0.9982
Methoxychlor	85	1.2	0.001	0.007	0.9982

## Results and discussion

### Concentration profile of OCPs in non-edible parts of plants

**Figure 2:**
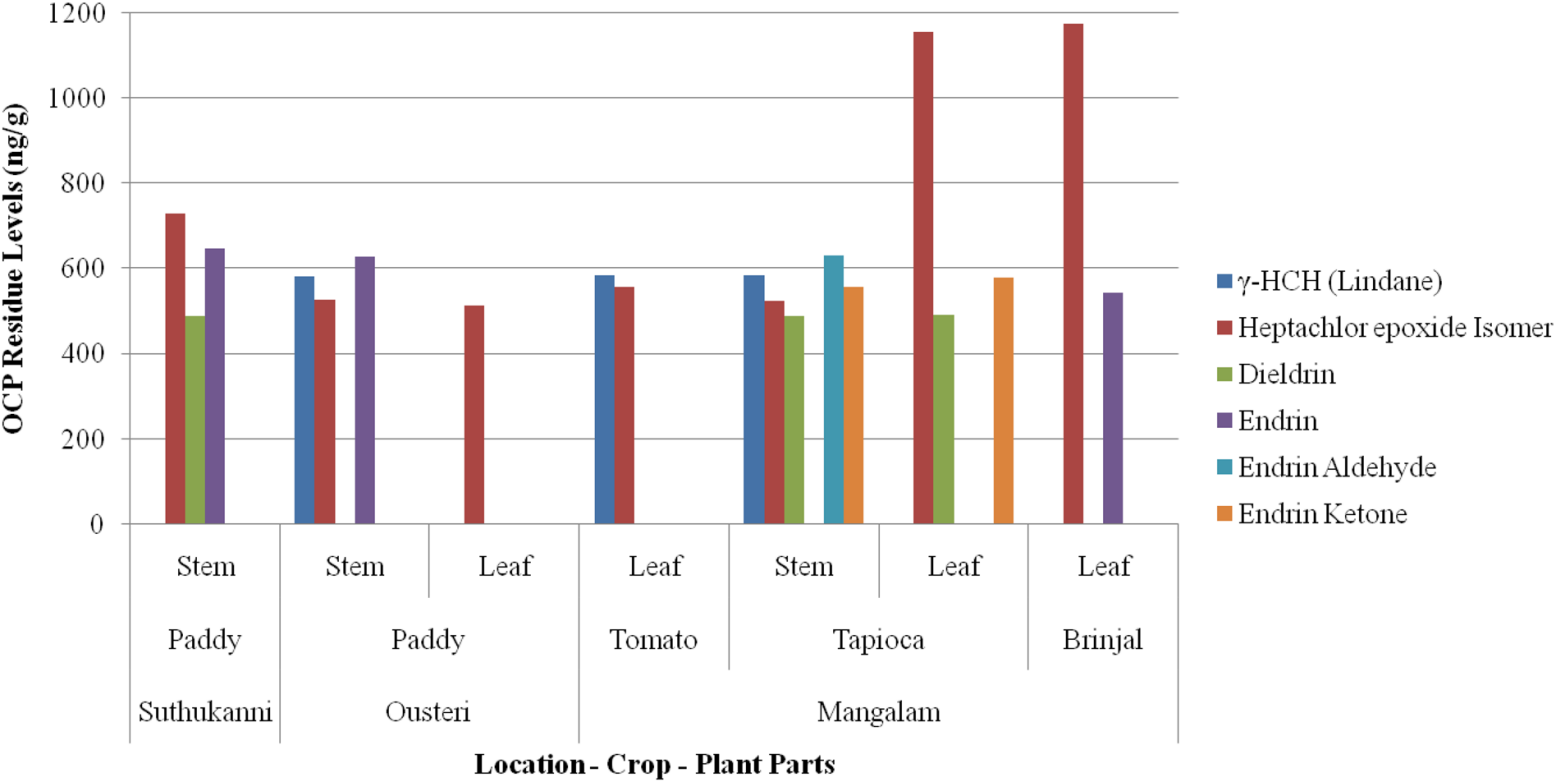
OCP residue levels in different plant samples.

GC-qMS data obtained for OCP residues were found in four varieties of plant samples, i.e. paddy, tomato, tapioca and brinjal from three locations i.e. Suthukanni, Ousteri and Mangalam in the study region (Fig. 2). Of the 18 OCP compounds analysed, residues of six compounds *viz*., *γ*-HCH (lindane), heptachlor epoxide isomer, dieldrin, endrin, endrin aldehyde and endrin ketone were found in seven plant samples (Fig. 2). The OCP residue concentrations in various parts of the plant species studied are discussed below.

#### Plant stem

*γ*-HCH (lindane) residues were found in paddy stem (581.14 ng/g) from Ousteri, and tomato leaf (583.3 ng/g) and tapioca stem (585.82 ng/g) both from Mangalam village. Heptachlor epoxide isomer was detected in seven plant samples and ranged from 512.53 to 1173.8 ng/g. Dieldrin was found in paddy stem (489.97 ng/g) from Suthukanni, tapioca stem (490.21 ng/g) and leaf (490.32 ng/g) from Mangalam. The Government of India’s Ministry of Agriculture has banned and restricted nearly 30 different types of pesticides, including some of the OCPs detected in this study, under the provisions of Insecticides Act, 1968. Among such chemicals, endrin was found in three samples with concentrations ranging from 542.39 ng/g in brinjal leaf (Mangalam village) to 647.96 ng/g in paddy stem Suthukanni village. Endrin aldehyde was detected in tapioca stem (629.88 ng/g), and endrin ketone in tapioca stem (557.03 ng/g) as well as leaf (578.65 ng/g) (Fig. 2).

#### Leaves

Leaves of all the plants (paddy, tomato, tapioca and brinjal) accumulated significant concentrations of OCPs (Fig. 2). Some of the OCPs such as endrin aldehyde and endrin ketone residues seem to have accumulated more in the stems of tapioca than other parts. The elevated levels in the stem and leaves could be attributed to the fact that most of the chemicals were sprayed on the aerial portion of the crop prior to the ripening season. Moreover, the growth stages of plants when these chemicals were applied can also be a contributing factor in their detections. Plants are contaminated with pesticide residues both directly (from application) and indirectly (by aerial drift, soil dust, volatilization and root uptake)^7^. Lipid and aqueous assisted distribution are responsible for moving the pesticides through the cuticle when applied to the plant shoots^39^. Generally, non-polar and undissociated compounds in the lipid soluble form pass through the cuticle, while the polar compounds enter through the aqueous route. Under conditions of elevated humidity both forms of compounds penetrate the cuticle. The plant-absorbed compounds may remain bound to the plant tissue, be stored in the vacuoles of the plant cells, form conjugates with the plant constituents, and also undergo degradation processes in the plant material. The unabsorbed materials on plant surface may be volatilized^8^, washed down from the leaf surface by the action of rainfall, or remain on the outer surface where it dries to crystalline form or concentrates to form a viscous liquid. Pesticides which are washed down are deposited in the soil and can be absorbed by the roots. The detection of OCPs in the leaf samples of paddy (rice), tomato, tapioca and brinjal could be attributed to phyllotaxy, wherein maximum portion of a leaf is exposed to the foliar spray of chemical pesticides.

#### Fruits/grain/vegetables

None of the OCP residues was found in fruit/grain/vegetables samples of paddy, tomato, tapioca and brinjal.

The order of highest concentration of OCP residues detected in the plant samples was heptachlor epoxide isomers > endrin > endrin aldehyde > γ-HCH > endrin ketone > dieldrin as shown in Fig. 2. Heptachlor epoxide isomers, endrin ketone and dieldrin were most frequently identified in leaves whereas endrin, endrin aldehyde and γ-HCH were found in stems. The levels of heptachlor epoxide isomer (512.53 ng/g and 1173.8 ng/g) detected in all the four plants samples were alarmingly high. All the OCPs detected were either from stem or leaves of the four crop/vegetable plants. Though these plant parts are generally not consumed by human beings, the chemical residues may find their way to land after disposal or to animals by direct ingestion, as the leaves are major fodder material for cattle and other livestock. Heptachlor epoxide isomer is known for its health consequences including endocrine disrupting functions in livestock and humans^40,41^. The other hazardous OCP residue detected was lindane. The half-life of lindane is 3 to 4 years whilst dieldrin is more persistent. Although India has banned the use of endrin in agriculture, considerable levels of endrin residues were detected in the samples, an apparent indication of illegal use of the chemical in farmlands. It may be noted that paddy is the predominant crop in the farmlands of Puducherry region. Paddy (rice) is one of the major food grains used in the region for their livelihood, hence utmost care should be taken and proper pesticide management plans should be implemented.

### Ecotoxicological impact of OCPs

Globally, the residue levels of pesticides are compared with their defined Maximum Residue Limits (MRL) in order to have an understanding on the extent of contamination and its associated toxicological impacts. The MRL is defined as the highest level of a pesticide residue that is legally permitted in or on food or feed when pesticides are applied correctly (in accordance with Good Agricultural Practice) and is established in the Codex Alimentarius of the FAO-WHO^42^. Moreover, country or region specific MRLs are also available for comparison like the one developed by European Commission (EU). The residue levels of the identified compounds in this study were significantly higher (Table 2) than the CODEX MRLs set for relevant category. In the present case the MRL levels as applicable for relevant category (feed), *viz*., straw and stems, are considered for comparison with the residue levels of OCPs. The current findings are alarming and represent the state of environmental contamination. All the six OCP compounds detected in the present study are enlisted as Persistent Organic Pollutants (POPs) under Annex-A of Stockholm Convention^43^, wherein the contracting parties must take measures to eliminate the production and use of such chemicals. In view of this, the reported levels of OCP residues in plant samples from farmlands in Puducherry region are of great environmental concern. These are also known carcinogens and recognized as endocrine disrupting chemicals (EDCs, Table 2). Because of the lipophilic nature of OCPs, plant materials with less water and more lipid contents are likely to accumulate the OCPs. The high concentration of OCP residues and their metabolites represents past signatures of its extensive use (chemical compounds and/or formulations) in the agricultural systems in and around Oussudu Lake. Because of the high toxicity and wider usage of pesticides, MRLs in food and feedstuff are developed by government and international organizations to ensure that the contaminants are not present at levels that could pose a health risk to the public and/or livestock. The present findings are indicative of the flow/movement of OCP residues to the biotic components of the ecosystem in the Puducherry region. Thus, the present study offers baseline information and emphasizes need to monitor the residues of OCPs in these habitats on a regular basis. On the whole, the findings of this study are the first reports of residue levels of OCPs in plants in farmlands in Puducherry region.

**Table 2:** Maximum Residue Levels (MRLs)^*^ for the OCP residues in plant samples.

Compound	Pesticide residue obtained (ppm) in the present study	Codex MRL (mg/kg )	Year of adoption	ADI (mg/kg BW)	Endocrine Disrupting Effects^$^
γ-HCH	0.581 – 0.586	0.01	2016	0–0.005	Reduction of oestrous cycles and luteal progesterone concentrations. Increase of insulin and estradiol blood serum concentrations, decrease thyroxine concentrations
Heptachlor epoxide isomer	0.513 – 1.174	NA	NA	NA	Binding to cellular estrogenic and androgen receptors
Dieldrin^1^	0.489–0.490	0.05–0.1	1997	0.0001	Competitive binding to androgen receptors, estrogenic effect, stimulation of estrogenic receptor production
Endrin^2^	0.542–0.648	0.05	1997	0.0002	Competitive binding to androgen receptors
Endrin Aldehyde	BDL–0.630	NA	NA	NA	
Endrin Ketone	0.557–0.579	0.05	1997	0.0002	

Source: http://www.fao.org/fao-who-codexalimentarius/standards/pestres/en/ (accessed on 09.03.2017). *MRLs as adopted by the Codex Alimentarius Commission up to and including its 39^th^ Session (July 2016). MRLs mentioned are for straw / stems, and these are based on extraneous residues.

^$^ Source: Munif et al. (2011).

^1^ 0.05 mg/kg is for leafy vegetables, and 0.1 mg/kg is for root and tuber vegetables.

^2^ for Fruiting vegetables.

*ADI* acceptable daily intake, *BW* body weight.

## Conclusions and recommendations

The present survey in Puducherry region reveals the use of various synthetic chemical pesticides in the farming system including the banned OCPs, i.e., endosulphan, polydol and DDT. Compounds, namely γ-HCH, heptachlor epoxide isomer, dieldrin, endrin, endrin aldehyde and endrin ketone were detected in brinjal, paddy, tapioca and tomato. The order of OCPs was heptachlor epoxide isomers > endrin > endrin aldehyde > γ-HCH > endrin ketone > dieldrin. Furthermore, heptachlor epoxide isomers, endrin ketone and dieldrin were most frequently detected in leaves whereas endrin, endrin aldehyde and γ-HCH were detected in stems. The only OCP detected in all the seven samples was heptachlor epoxide isomer, with its residue concentration range of 512.53–1173.8 ng/g. The levels of all the six OCPs detected were 10–50 times higher than the MRLs set in FAO/WHO’s CODEX Alimentarius for its consumption under appropriate category. Nevertheless, considering the topographical features of the study area and its strategic location, it is suggested that regular monitoring of residues of OCPs be carried out to understand the toxicological impacts on Oussudu Lake in general and the entire Puducherry region at large scale. Since the lake is spread over both Pondicherry and Tamil Nadu states, a similar survey and monitoring is required to be undertaken in villages on Tamil Nadu side, which will help in devising a comprehensive strategy for environmental monitoring of chemical residues in different environmental segments, jointly by both the states. The findings of the present study will aid in implementing effective environmental mitigation plans in the region.
